# Patterns of Antibody Binding to Aquaporin-4 Isoforms in Neuromyelitis Optica

**DOI:** 10.1371/journal.pone.0010455

**Published:** 2010-05-05

**Authors:** Simone Mader, Andreas Lutterotti, Franziska Di Pauli, Bettina Kuenz, Kathrin Schanda, Fahmy Aboul-Enein, Michael Khalil, Maria K. Storch, Sven Jarius, Wolfgang Kristoferitsch, Thomas Berger, Markus Reindl

**Affiliations:** 1 Clinical Department of Neurology, Innsbruck Medical University, Innsbruck, Austria; 2 Department of Neurology, SMZ-Ost Donauspital, Vienna, Austria; 3 Department of Neurology, Medical University of Graz, Graz, Austria; 4 Division of Molecular Neuroimmunology, Department of Neurology, University of Heidelberg, Heidelberg, Germany; Julius-Maximilians-Universität Würzburg, Germany

## Abstract

**Background:**

Neuromyelitis optica (NMO), a severe demyelinating disease, represents itself with optic neuritis and longitudinally extensive transverse myelitis. Serum NMO-IgG autoantibodies (Abs), a specific finding in NMO patients, target the water channel protein aquaporin-4 (AQP4), which is expressed as a long (M-1) or a short (M-23) isoform.

**Methodology/Principal Findings:**

The aim of this study was to analyze serum samples from patients with NMO and controls for the presence and epitope specificity of IgG and IgM anti-AQP4 Abs using an immunofluorescence assay with HEK293 cells expressing M-1 or M-23 human AQP4. We included 56 patients with definite NMO (n = 30) and high risk NMO (n = 26), 101 patients with multiple sclerosis, 27 patients with clinically isolated syndromes (CIS), 30 patients with systemic lupus erythematosus (SLE) or Sjögren's syndrome, 29 patients with other neurological diseases and 47 healthy controls. Serum anti-AQP4 M-23 IgG Abs were specifically detected in 29 NMO patients, 17 patients with high risk NMO and two patients with myelitis due to demyelination (CIS) and SLE. In contrast, IgM anti-AQP4 Abs were not only found in some NMO and high risk patients, but also in controls. The sensitivity of the M-23 AQP4 IgG assay was 97% for NMO and 65% for high risk NMO, with a specificity of 100% compared to the controls. Sensitivity with M-1 AQP4 transfected cells was lower for NMO (70%) and high risk NMO (39%). The conformational epitopes of M-23 AQP4 are the primary targets of NMO-IgG Abs, whereas M-1 AQP4 Abs are developed with increasing disease duration and number of relapses.

**Conclusions:**

Our results confirm M-23 AQP4-IgG Abs as reliable biomarkers in patients with NMO and high risk syndromes. M-1 and M-23 AQP4-IgG Abs are significantly associated with a higher number of relapses and longer disease duration.

## Introduction

Neuromyelitis optica (NMO) is a demyelinating neurological disease defined by optic neuritis (ON) and longitudinally extensive transverse myelitis (LETM) [1], [2]. NMO often leads to severe disability and even death within several years of disease onset [1], [3]. Since the discovery and validation of NMO-IgG serum antibodies (Abs) in NMO patients [4], [5], NMO is considered to be a separate disease entity to multiple sclerosis (MS) [6], [7], [8], [9]. Compared to MS, NMO patients have a worse prognosis and require different treatment strategies according to the dominant humoral immunopathogenesis in NMO. Thus, early discrimination from MS enables specific attention for and treatment of NMO patients [10], [11], [12], [13]. The specificity of NMO-IgG Abs for the disease led to addition of NMO-IgG Abs to the diagnostic criteria of NMO [14]. NMO-IgG are especially useful in the early phase of disease after a first episode of LETM or recurrent ON. More than half of NMO-IgG seropositive patients with first LETM relapse within half a year [15]. NMO-IgG Abs have also been detected in patients with non organ specific autoimmunity such as in systemic lupus erythematosus (SLE) or Sjögren syndrome (SS) patients [16]. NMO-IgG Abs target AQP4 [17], the predominant water-channel protein within the central nervous system (CNS) [18]. AQP4 exists as two different heterotetramers [19], M-1 and M-23 AQP4, which result from usage of different start codons [20], [21] and vary in the 23 amino acids in the N terminus of the protein [19]. Contrary to full length AQP4, M-23 AQP4 forms orthogonally arranged particles (OAPs) [20], which were shown to be potential targets for antibody binding [20], [22].

Although AQP4 antibodies have now been analyzed in several cohorts of NMO patients worldwide and the importance of AQP4 OAPs has been demonstrated in all of these studies, it is not clear whether the specificity and sensitivity of the antibody response to AQP4 differs between these two isoforms. To the best of our knowledge no systematic study has so far analyzed the immune response to both AQP4 M-1 and M-23 isoforms in NMO and high risk NMO and their follow-up samples. We therefore screened serum probes of patients with NMO, MS, clinically isolated syndromes (CIS), other neurological diseases (OND), SLE and healthy controls (HC) for M-1 and M-23 AQP4-IgG and- IgM. We were also interested to compare clinical characteristics of patients showing the antibody response and, in addition, to assess the value of anti-AQP4 IgM antibodies in our cohort.

## Materials and Methods

### Patients and serum samples

Serum samples from 30 patients with NMO and 26 patients with high risk NMO were recruited prospectively from 2007 to 2009 by the Austrian NMO Study-Group from several Austrian Neurological Departments, or were sent in for AQP4 antibody testing by the Department of Neurology, University of Heidelberg, Germany (n = 10). The Austrian NMO Study-Group was established to obtain clinical, neuroradiological and immunological data of Austrian patients with definite and high risk NMO, to enable an early and appropriate treatment, and to determine the so far unknown prevalence of NMO in Austria. The present study was approved by the ethical committee of Innsbruck Medical University (study no. UN3041 257/4.8) and all Austrian patients gave written informed consent to the study protocol. All German samples were tested in an anonymized fashion as requested by the institutional review board of the University of Heidelberg. All NMO patients met the revised diagnostic criteria of 1999 [1] and 97% of patients showed longitudinally extensive transverse myelitis extending over more than three vertebral segments. Ninety-seven percent of definite NMO cases were females (Table 1). The high risk group of NMO patients comprised two patients with recurrent ON (8%) and 24 patients with a single episode or recurrent LETM (92%), including three neuropsychiatric SLE patients and two patients with neurosarcoidosis. Additionally, we included 101 patients with MS according to the revised “McDonald Criteria” [23]: 64 patients with relapsing remitting MS (RRMS), 13 patients with primary progressive MS (PPMS) and 24 patients with secondary progressive MS (SPMS). Moreover, 27 patients with CIS, 29 patients with various OND (ischemic infarct, parkinson disease, epileptic seizure, radiculopathy, insomnia, sleep apnoea syndrome, CNS lymphoma, traumatic brain injury, myasthenia gravis, chronic inflammatory demyelinating polyneuropathy, vestibular neuritis, orthostatic syncope, psychogenic neurological symptoms, CNS vasculitis, hereditary neuropathy, analgesic-induced headache, neuroborreliosis, viral encephalitis, chronic tension-type headache, glioblastoma multiforme), 30 patients with SLE or SS and 47 HC were screened for AQP4-Ig.

**Table 1:** Serum antibody binding AQP4 M-1 and M-23 isoforms in different disease groups.

AQP4	NMO	High risk NMO	MS	CIS	SLE/SS	OND	HC	p-value
Number	30	26	101	27	30	29	47	
Females	29 (97%)	16 (62%)	65 (64%)	21 (78%)	27 (90%)	20 (69%)	39 (83%)	0.001 ^2^
Age (y) 1	49 (18–80)	49 (26–75)	40 (16–68)	35 (19–63)	41 (22–92)	44 (18–84)	43 (21–68)	0.001 ^3^
M-23 IgG	29 (97%) *#	17 (65%) *	0 (0%)	1 (4%)	1 (3%)	0 (0%)	0 (0%)	<0.001 ^2^
M-23 IgG Titer (1:) 1	2,560 (160–20,480)	1,280 (40–10,240)	-	640	320	-	-	
M-1 IgG	21 (70%) *	10 (39%) *	0 (0%)	1 (4%)	0 (0%)	0 (0%)	0 (0%)	<0.001 ^2^
M-1 IgG Titer (1:) 1	160 (40–5,120)	80 (20–2,560)	-	40	-	-	-	
M-23 IgM	8 (27%) *	3 (12%)	4 (4%)	1 (4%)	0 (0%)	1 (4%)	0 (0%)	<0.001 ^2^
M-23 IgM Titer (1:) 1	40 (20–80)	80 (20–80)	50 (20–80)	20	-	20	-	
M-1 IgM	3 (10%)	2 (8%)	0 (0%)	0 (0%)	0 (0%)	0 (0%)	0 (0%)	0.001 ^2^
M-1 IgM Titer (1:) 1	40 (20–40)	30 (20–40)	-	-	-	-	-	

1 data are shown as median (range), p-value: groups were compared using ^2^ Chi-Square test and ^3^ Kruskal-Wallis test and Dunn's multiple comparison post-hoc test,

*statistically different from HC group at p<0.01,

# statistically different from high risk NMO group at p<0.01.

Abbreviations: n  =  number of patients, y  =  years.

Serial blood samples were available from two patients with recurrent ON who converted to NMO after 2.6 and 8.7 years.

None of the patients was under high-dose methylprednisolone treatment (HDMP) at the time of blood sampling.

All samples were stored at −20°C until use.

### Expression of AQP4 in HEK-293A cells

Human M-1 and M-23 AQP4 isoforms were amplified from a human adult spinal cord Quick Clone^Tm^ cDNA library (Clontech-Takara Bio Europe, Saint-Germain-en-Laye, France) and cloned into the mammalian expression vector Vivid Colours™ pcDNA™ 6.2C-EmGFP-GW/TOPO (Invitrogen, Carlsbad, CA, USA). The correct M-1 and M-23 AQP4 insert sequences were verified by DNA sequencing (Microsynth, Balgach, Switzerland). HEK-293A cells (ATCC, LGC Standards GmbH, Wesel, Germany) were transiently transfected (Fugene 6 transfection reagent, Roche Applied Sciences, Mannheim, Germany) achieving over-expression of AQP4 isoforms fused C-terminally to emerald green fluorescence protein (EmGFP), respectively. The empty vector served as control. Furthermore, both AQP4 isoforms were cloned into the pcDNA3.1 Directional TOPO Expression vector (Invitrogen, Carlsbad, CA, USA), to express M-1 and M-23 AQP4 without the EmGFP fusion protein.

Efficiency of transfection was determined by flow cytometry (FACScan and Cell Quest pro software, BD Biosciences, San Jose, CA, USA). The topology of AQP4 was determined via intracellular staining using a rabbit anti-AQP4 Ab detecting amino acids 249–323 (Sigma-Aldrich). Cells were fixed with 4% paraformaldehyde, blocked with goat IgG (Sigma-Aldrich), incubated with the antibody, washed with phosphate buffered saline (PBS)/10% FCS and incubated with Cy^Tm^3-conjugated AffiniPure Goat anti-rabbit IgG (Jackson ImmunoResearch Laboratory, West Grove, PA).

### AQP4-Ig Immunofluorescence (IF) assay

All serum samples were analyzed for the presence of AQP4-IgG and- IgM by an extracellular live cell staining IF technique as previously described [24], [25], [26].

HEK-293A cells were transfected using the expression plasmids mentioned above and cultured for 72 h. Then, cells were blocked with goat IgG (Sigma-Aldrich) in PBS/10%FCS, incubated with pre-absorbed (rabbit liver powder, Sigma-Aldrich) serum samples (screening dilution 1∶20 and 1∶40, positive sera were further diluted until loss of signal), washed and detected with Cy^Tm^3-conjugated goat anti-human IgG or IgM antibody (Jackson ImmunoResearch Laboratory, West Grove, PA). Dead cells were visualized with DAPI staining (Sigma-Aldrich) and live cells were analyzed for AQP4-Ig binding. All samples were assessed by two independent investigators blinded for any clinical information. Anti-AQP4 antibodies purified from a NMO patient's plasma by affinity chromatography served as positive control [25].

### Statistical analysis

Statistical analysis (means, medians, range, standard deviations), significance of group differences and linear regression were evaluated using SPSS software (release 16.0, SPSS Inc., USA) or GraphPad Prism 5 (GraphPad, San Diego, USA). Between-group comparisons were performed with Kruskal-Wallis test, Dunn's multiple comparison post-hoc test, Mann-Whitney *U* test, Fisher's exact test and Chi-square test. Correlation of parameters was analyzed with Spearman's non-parametric correlation. Statistical significance was defined as two-sided p-value <0.05 and Bonferroni corrections were applied for multiple comparisons when appropriate.

## Results

### M-1 and M-23 AQP4-IgG binding results in different staining patterns

With the advent of AQP4 Abs as biomarkers in NMO [4], [17], various NMO-IgG antibody assays have been developed so far. In our study we screened serum samples by a live cell staining IF assay, based on the publication of Takahashi et al [24].

**Figure 1:**
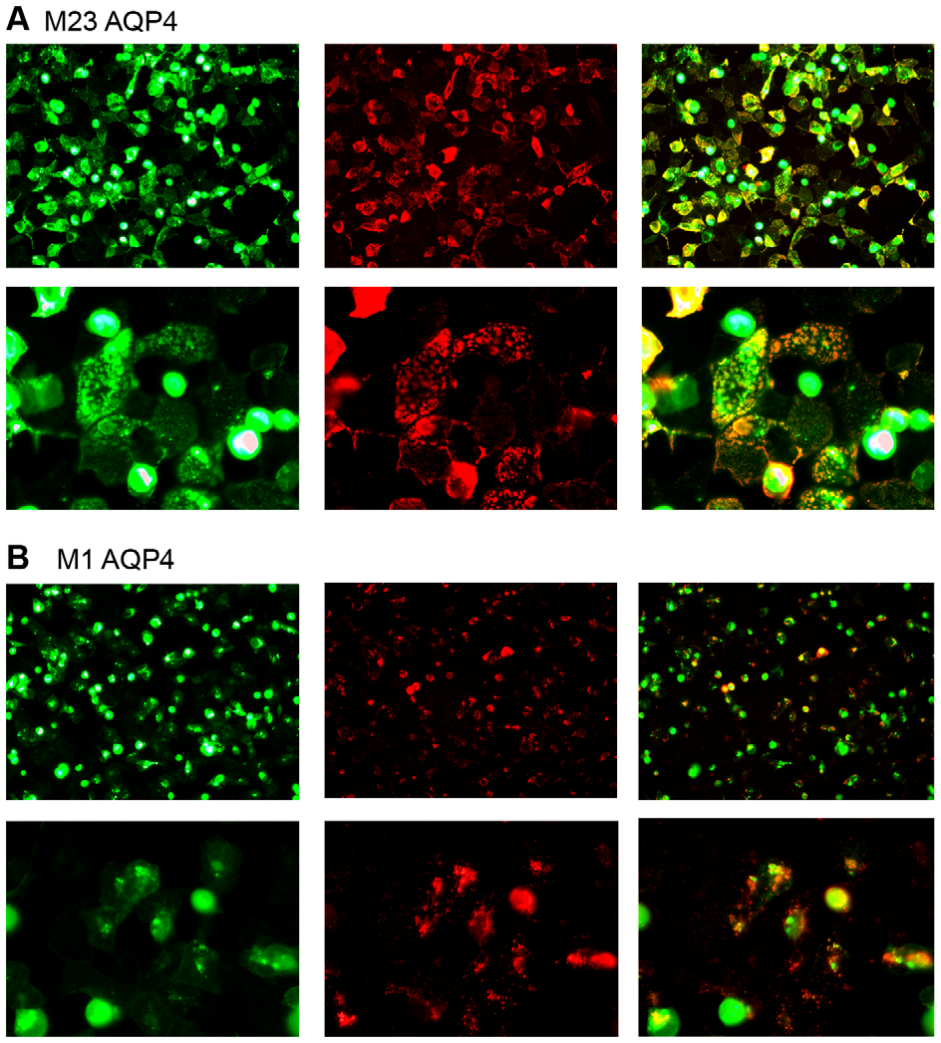
Different staining patterns of NMO-IgG in M-1 and M-23 AQP4 transfected cells. Anti-AQP4 IgG (red) in NMO patient's serum targets AQP4 (green), which is expressed by transiently transfected HEK cells. Performing the assay for M-23 AQP4 (**A**, green) versus M-1 AQP4 (**B**, green), results in different staining patterns of NMO-IgG (red). Weaker binding was observed to M-1 AQP4, which contrary to M-23 AQP4 forms only few orthogonal arrays of particles.

In a first step we verified the AQP4 topology of transfected cells with the rabbit anti-water channel AQP4 antibody, followed by the determination of the transfection efficiency via flow cytometry staining (EmGFP 68±5%; M-23 AQP4 64±2%; M-1 AQP4 58±5%; statistically non-significant). We then analyzed the presence of anti-AQP4 Abs in patients' sera by our immunofluorescence assay. As shown in Figure 1 we found co-localization of NMO Abs (red) with the AQP4 protein (green), whereas NMO Abs did not bind to EmGFP transfected cells. All serum samples were screened for the presence of AQP4 M-23 (Figure 1A) and M-1 IgG (Figure 1B), which resulted in different staining patterns. Antibody binding to M-23 AQP4 showed a laminar staining pattern, which could resemble the formation of OAPs. In contrast, M-1 AQP4 did not form visible OAPs and therefore had a more point shaped staining pattern (Figure 1).

**Figure 2:**
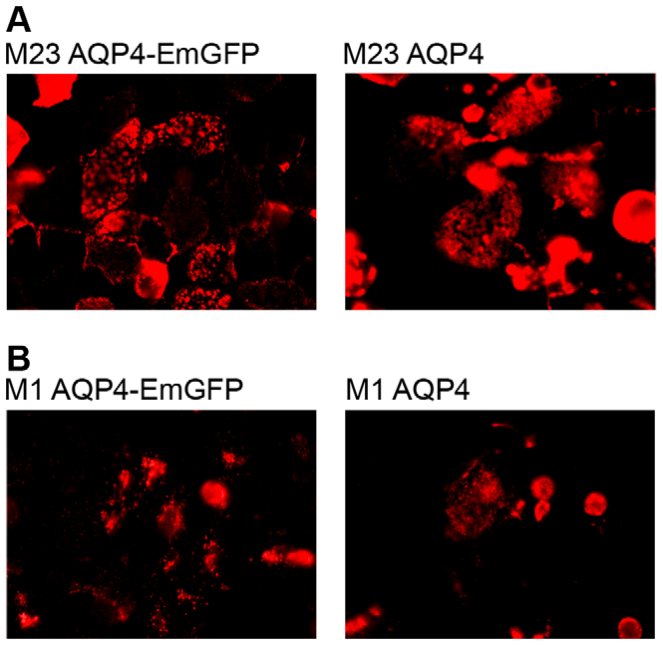
NMO-IgG staining patterns in AQP4-EmGFP versus AQP4 expressing cells. Fusion of EmGFP to AQP4 molecules has no effect on the formation of the different staining patterns of NMO-IgG in M-1 and M-23 AQP4 transfected cells. NMO-IgG has the same laminar staining pattern when binding M-23 AQP4 with and without EmGFP fusion (A). In contrast, cells transfected with M-1 AQP4-EmGFP and M-1 AQP4 have a more point shaped staining pattern (B).

In order to exclude that EmGFP might influence the formation of large arrays compared to the non-fused AQP4 proteins we have also over-expressed M-1 and M-23 proteins without EmGFP. We observed the same NMO-IgG binding patterns using M-1 and M-23 AQP4 transfected cells without the EmGFP fusion protein, as illustrated in Figure 2. Consequently we can exclude that EmGFP has an influence on the formation of OAPs. Furthermore, EmGFP fused versus unfused AQP4 M-1 and M-23 transfected cells gave identical results in a subset of 15 M-1 and M-23 seropositive and 15 M-1 and M-23 seronegative patients.

### NMO-IgG mainly targets M-23 AQP4

Anti-AQP4 M-23 specific IgG was detected in 29 of 30 definite NMO patients and in 17 of 26 patients with high risk NMO (Table 1). Thus the sensitivity of our assay using M-23 AQP4 transfected cells was 97% for NMO patients and 65% for patients with suspected NMO. In the high risk NMO group, three patients with SLE associated LETM and one patient with recurrent ON were seropositive for anti-AQP4 M-23 IgG. Additionally, M-23 AQP4-IgG was detected in two patients with myelitis due to demyelination (CIS) and SLE, respectively. Furthermore, all HCs, patients with MS, OND and non-neuropsychiatric SLE patients were seronegative for anti-AQP4 M-1 and M-23 IgG. In contrast, sensitivity with M-1 AQP4 transfected cells was remarkably lower for clinically definite NMO (70%) and high risk NMO (39%). Additionally, serum Abs to full length AQP4 were also detected in the patient with isolated myelitis, but not in the patient with SLE-myelitis, who were both positive for M-23 AQP4-IgG.

To summarize, weaker binding was observed to full length AQP4, which is also evident from the lower titer values of M-1 AQP4-IgG (Table 1).

### NMO and high risk patients can be classified into three groups

According to their NMO-IgG serostatus, we classified definite and high risk NMO patients into three different groups (Table 2): 1) seronegative patients (n = 10); 2) patients with Abs against M-23 AQP4 but not against full length AQP4 (n = 15); and 3) patients with Abs against both AQP4 isoforms (n = 31). As described before, AQP4-IgG were predominantly detected in the sera of female patients: 93% of patients with M-23 AQP4-IgG, 94% of cases with M-1 and M-23 AQP4-IgG, but only 20% of seronegative patients.

**Table 2:** Different features of NMO and High risk patients according to their AQP4 Ab status.

	M1-M23-	M1-M23+	M1+M23+	p-value
Number	10	15	31	
Females	2 (20%)	14 (93%) *	29 (94%) *	<0.001 ^2^
Age (y) 1	40 (26–65)	54 (18–77)	49 (19–80)	0.095 ^3^
Duration (m) 1	8 (0.8–60)	45 (0.1–114)	70 (0.4–540) *#	0.009 ^3^
NMO	1 (10%)	8 (53%)	21 (68%) *	0.006 ^2^
Myelitis	9 (90%)	15 (100%)	30 (97%)	0.413 ^2^
LETM	9 (90%)	15 (100%)	29 (94%)	0.179 ^2^
ON	2 (20%)	8 (53%)	22 (71%) *	0.026 ^2^
Cerebral MRI	0 (0%)	5 (33%)	8 (26%)	0.131 ^2^
OCBs	2 (20%) ^5^	2 (13%) ^6^	2 (7%) ^7^	0.675 ^2^
Relapses 1	1 (0–4)	4 (1–13) *	6 (1–15) *	<0.001 ^3^
M-23 IgG Titer 1	-	640 (40–10,240)	2,560 (160–20,480) #	<0.001 ^4^
M-1 IgG Titer 1	-	-	160 (20–5,120)	

1 data are shown as median (range), p-value: groups were compared using ^2^ Chi-Square test and ^3^ Kruskal-Wallis test and Dunn's multiple comparison post-hoc test,^ 4^ Mann-Whitney *U* test * statistically different from M1-M23- group at p<0.01, # statistically different from M1-M23+ group at p<0.01. data available of ^5^ 8, ^6^ 13 and ^7^ 23 patients.

Abbreviations: n  =  number of patients, y  =  years, m  =  months.

Whereas only one NMO patient was seronegative for AQP4-IgG, the majority of patients with Abs against both AQP4 isoforms (68%) and with Abs against M-23 AQP4 (53%) were diagnosed as NMO. We found no significant differences in the percentage of patients with myelits, LETM, ON, cerebral MRI lesions and oligoclonal bands in the three groups.

M-1 AQP4-IgG seropositivity is significantly associated with a longer median disease duration (70 months), compared to patients who were only positive for M-23 AQP4-IgG (45 months) and the subjects without AQP4 Abs (8 months). Additionally, the median number of relapses was higher in the M-1 and M-23 AQP4-IgG double positive group (6) and in the M-1 negative but M-23 AQP4-IgG positive group (4) than in the seronegative patients (1).

Furthermore, median antibody titers to M-23 AQP4 were significantly higher in the M-1+M-23 AQP4-IgG double positive group (1∶2,560) than those cases only positive for M-23 AQP4-IgG (1∶640).

**Figure 3:**
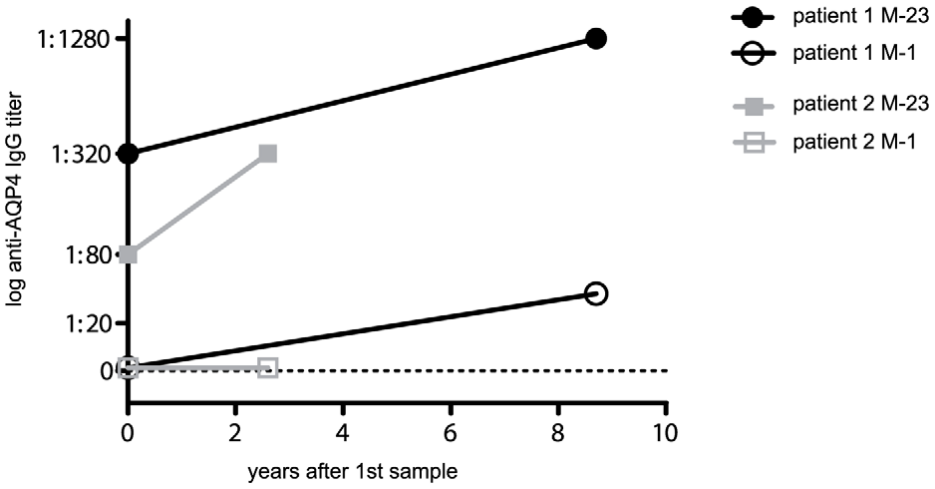
M-1 and M-23 AQP4-IgG titer values in follow-up samples. Higher titer values of NMO-IgG in two patients with recurrent ON (1^st^ sample) after conversion into NMO (2^nd^ sample) after 2.6 and 8.7 years. With increasing M-23 AQP4-IgG titers, patient one developed Abs against full length AQP4, whereas patient two remained M-1 AQP4-IgG negative.

Additionally, our results show an increase of NMO-IgG titers in the serum of two patients with recurrent ON after converting into definite NMO (Figure 3). With increasing M-23 AQP4-IgG titers, the patient with the longer disease duration developed Abs against full length AQP4, whereas the second patient remained M-1 AQP4-IgG negative.

### NMO-IgM is elevated in definite and high risk NMO patients

A higher percentage of M-23 IgM Abs was detected in NMO patients (n = 8; 27%) and in the high risk group (n = 3; 12%) compared to the other groups (Table 1). M-23 IgM Abs were also present in the NMO-IgG seropositive patient with isolated myelitis, in four patients with MS (4%) and in one OND patient (4%).

Furthermore, anti-AQP4-IgM Abs to M-1 AQP4 were only detectable in a small subgroup of patients with definite NMO (n = 3) and high risk NMO (n = 2). In contrast to NMO-IgG, AQP4-IgM positive samples yield much lower titer values (Table 1).

## Discussion

Our assay with living AQP4 M-23 transfected cells is highly sensitive for detecting anti-AQP4-IgG Abs in definite (97%) and high risk (65%) NMO patients. Furthermore, we detected M-23 AQP4-IgG in two patients with myelitis (CIS and SLE), who harbour the risk of later developing NMO and could benefit from an appropriate early treatment. In contrast, AQP4-IgG Abs were not present in patients with MS, OND, and non-neuropsychiatric SLE and in HC, which supports their high relevance as biological markers. Our results are in concordance with studies showing highest sensitivity for NMO-IgG detection in cell based assays [27].

Recent papers suggest stronger NMO-IgG Ab binding directed to the shorter M-23 AQP4 isoform [22], which in contrast to M-1 AQP4 can form orthogonal arrays (OAPs) [20], [28]. Our study confirms weaker binding of NMO-IgG to full length AQP4, resulting in a lower sensitivity for clinically definite NMO (70%) and high risk NMO (39%) patients, besides also the M-23 IgG seropositive patient with SLE associated myelitis was negative for full length AQP4-IgG. Consequently the assay with M-23 AQP4 transfected cells is much more reliable for an early identification of NMO-IgG Abs in serum samples, whereas numerous patients turned out to be “false negative” when performing the assay with M-1 AQP4 transfected cells.

NMO-Ig Abs resulted in different staining patterns when binding to full length AQP4 in contrast to the M-23 AQP4 isoform. We speculate that the laminar staining pattern of M-23 AQP4-IgG is due to the capability of M-23 AQP4 to form OAPs, which are suggested to be potential targets for Ab binding. On the contrary, IgG binding to full length AQP4, resulted in a more point shaped staining pattern. In order to exclude the possibility that EmGFP has an influence on the formation of OAPs, we performed the immunofluorescence assay with both AQP4 isoforms without EmGFP fusion protein, and obtained same staining patterns and identical results for serum M-1 and M-23 AQP4-IgG and- IgM seropositive and seronegative samples.

As recent studies suggest a strong correlation of NMO-IgG titres with the clinical status of disease [24], [29] we performed serial dilutions of NMO Ig positive samples, resulting in higher titer levels of M-23 AQP4-IgG. Moreover, none of the patients were only positive for M-1 AQP4-IgG and negative for M-23 AQP4-IgG, or had higher titer levels for full length AQP4. Possibly, the differences in sensitivity and specificity of the various published NMO-IgG Ab assays could be due to the AQP4 isoform [30], in particular earlier papers do not differentiate between M-1 versus M-23 AQP4-IgG. According to their AQP4-IgG serostatus, we could classify definite and high risk NMO patients into three different groups: AQP4-IgG seronegative patients, patients with Abs against M-23 AQP4 but not against full length AQP4 and patients with Abs against both AQP4 isoforms.

Our results indicate a primary NMO-IgG response directed to an epitope present in the shorter M-23 AQP4 isoform, which could be an epitope present in OAPs. It seems, however, that with increasing disease duration and severity, patients develop higher titers for M-23 AQP4-IgG and additionally target epitopes present in full length AQP4, thus also M-1 AQP4-IgG titer values increase with time. This hypothesis is supported by the significant higher number of relapses and the longer disease duration in patients with M-1 and M-23 IgG compared to patients who target only M-23 AQP4. Moreover, AQP4-IgG negative patients (definite and high risk) had the shortest disease duration and lowest number of relapses. Alternatively, this finding could also be explained by an affinity maturation of these antibodies like it has been shown for other high-affinity IgG autoantibodies that have undergone somatic hypermutation and class switching thus reflecting a pathologic process [31].

The difference in titer levels was also seen in two patients who were initially diagnosed with recurrent ON. The M-23 AQP4-IgG titer was much higher after they had developed definite NMO, and the patient with longer disease duration developed Abs against M-1 AQP4.

In comparison to AQP4-IgG, AQP4-IgM Abs are not a reliable biomarker, although they were elevated in definite and high risk NMO patients. However, AQP4-IgM were also detected in a low percentage of patients with MS and OND.

In this study we confirm that AQP4-IgG are useful biomarkers for early diagnosis of NMO and high risk patients. Our results suggest M-23 AQP4 as initial and major target antigen for antibody binding in definite and high risk NMO patients, whereas Abs to M-1 AQP4 are predominantly developed with increasing disease duration and severity. Thus we suggest that only M-23 AQP4 should be used as target antigen for the early detection of AQP4-IgG. AQP4-IgM was elevated in AQP4-IgG positive patients, however because of lower sensitivity and specificity its role as biomarker in NMO remains unclear.
